# Foot-and-Mouth Disease in Bolivia: Simulation-Based Assessment of Control Strategies and Vaccination Requirements

**DOI:** 10.1155/tbed/9055612

**Published:** 2025-09-25

**Authors:** Nicolas C. Cardenas, Diego Viali dos Santos, Daniel Magalhães Lima, Hernán Oliver Daza Gutierrez, Daniel Rodney Gareca Vaca, Gustavo Machado

**Affiliations:** ^1^Department of Population Health and Pathobiology, College of Veterinary Medicine, North Carolina State University, Raleigh, North Carolina, USA; ^2^Pan American Center for Foot-and-Mouth Disease and Veterinary Public Health, Pan American Health Organization, Rio de Janeiro, Brazil; ^3^Servicio Nacional de Sanidad Agropecuaria e Inocuidad Alimentaria (SENASAG), Santa Cruz de la Sierra, Bolivia

**Keywords:** dynamical models, epidemiology, infectious disease control, targeted control, transmission

## Abstract

Examining the dissemination dynamics of foot-and-mouth disease (FMD) is critical for revising national response plans. We developed a stochastic susceptible-exposed-infected-recovered (SEIR) metapopulation model to simulate FMD outbreaks in Bolivia and explore how the national response plan impacts the dissemination among all susceptible species. We explored variations in the control strategies, mapped high-risk areas, and estimated the number of vaccinated animals during the reactive ring vaccination. Initial outbreaks ranged from 1 to 357 infected farms, with control measures implemented for up to 100 days, including control zones, a 30-day movement ban, depopulation, and ring vaccination. Combining vaccination (50–90 farms/day) and depopulation (1–2 farms/day) controlled 60.3% of outbreaks, while similar vaccination but higher depopulation rates (3–5 farms/day) controlled 62.9% and eliminated outbreaks 9 days faster. Utilizing depopulation alone controlled 56.76% of outbreaks, but had a significantly longer median duration of 63 days. Combining vaccination (25–45 farms/day) and depopulation (6–7 farms/day) was the most effective approach, eliminating all outbreaks within a median of 3 days (with a maximum of 79 days). Vaccination alone controlled only 0.6% of outbreaks and had a median duration of 98 days. Ultimately, results showed that the most effective strategy involved ring vaccination combined with depopulation, requiring a median of 925,338 animals to be vaccinated. Outbreaks were most frequent in high-density farming areas, such as Potosí, Cochabamba, and La Paz. Our results suggest that emergency ring vaccination alone cannot eliminate FMD if reintroduced in Bolivia, and combining depopulation with vaccination significantly shortens the outbreak duration. These findings provide valuable insights to inform Bolivia's national FMD response plan, including vaccine requirements and the role of depopulation in controlling outbreaks.

## 1. Introduction

Foot-and-mouth disease (FMD) is a contagious viral disease that impacts cloven-hoofed animals, such as cattle, swine, small ruminants, and wildlife [1]. In the United Kingdom and the Netherlands during the 2001 FMD outbreaks, more than 6.7 million animals were slaughtered, including healthy sheep, goats, and pigs, as a precautionary measure [2, 3], generating substantial economic consequences given animal loss, decreasing animal milk and beef production and affecting other industries, such as tourism, with estimated costs ranging from 2.7 to 3.2 billion [4].

In recent years, South American countries have significantly advanced FMD control and eradication, achieving notable results [5, 6], for instance, Bolivia has made remarkable progress in controlling FMD, as of 2024, Bolivia, including the Department of Beni and the northern part of the Department of La Paz, has been recognized as the latest FMD-free zone. Bolivia stopped preventative vaccination in 2023 to become an FMD-free country. In contrast, the last outbreaks occurred in Colombia in 2017 and 2018 [7], since then, no outbreaks have been officially reported in Latin America, despite Venezuela's not achieving FMD-free status by WOAH [5]. However, the reintroduction of FMD remains a significant threat given the susceptibility of the cloven animal population to FMD in Latin America, as evidenced by the massive 2001 outbreak that affected 2027 farms in Uruguay [8].

Mathematical models have been used to simulate FMD outbreaks, allowing for effective testing control and elimination plans' effectiveness [9, 10]. Simulation models have also been used to identify outbreak spatial dissemination and estimate the number of vaccine doses, and estimate the depopulation capacity [11, 12]. Mechanistic mathematical modeling, provides valuable insights into how the disease may unfold under various conditions, allowing animal health authorities to plan accordingly for outbreak potential and estimate necessary resources [13–15].

We begin with analyzing the Bolivian national livestock farms' spatial distribution and animal movement data analysis to determine species densities by regions used in selecting farms for seed infection. We then extended our multihost, single-pathogen, multiscale model MHASpread model presented by Cardenas et al. [14] to (i) simulate the spread of FMD in Bolivia and evaluate the effectiveness of various control strategies, (ii) map the spatial distribution patterns of outbreaks, and (iii) based on the results, estimate the number of vaccinated animals under each scenario conditions simulated.

## 2. Materials and Methods

### 2.1. Population Data

**Figure 1:**
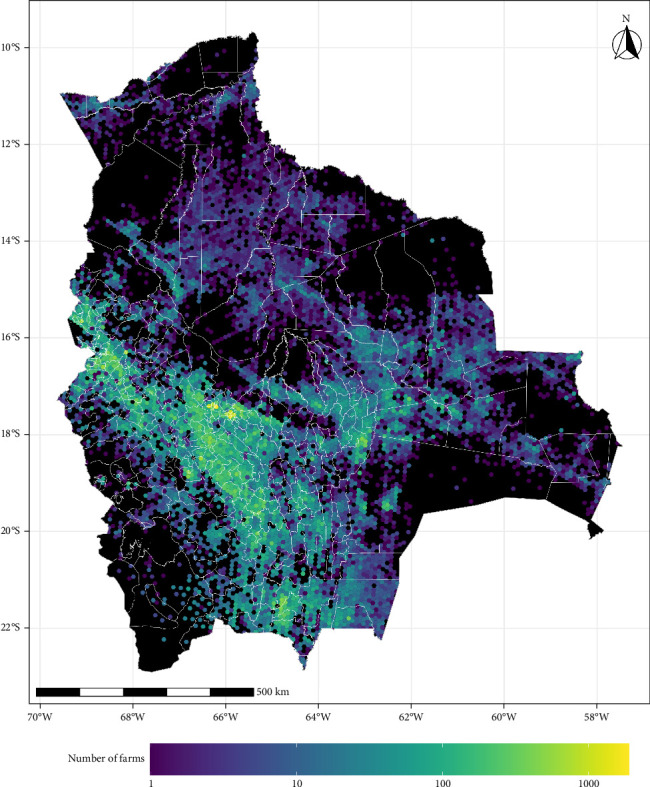
Bolivia's livestock density distribution. 10 km^2^ hexagon polygon with total livestock population overlayed with municipalities (white borders).

We used data from the Bolivian livestock farms from January 1, 2023, to July 25, 2024, provided by the Servicio Nacional de Sanidad Agropecuaria e Inocuidad Alimentaria [16] in Bolivia. The dataset included information on 218,143 farms, 183,752 cattle, 309 buffalo, 70,083 swine, and 123,011 sheep and goats. We grouped cattle and buffalo farms into one group named hereafter as “bovines,” while sheep and goat farms were categorized as “small ruminants.” After excluding 4034 farms (1.8%) due to missing geographical coordinates or number of heads, the final dataset consisted of 214,109 farms (Figure 1), 169,426 bovine farms, 53,313 swine farms, and 121,112 small ruminant farms.

### 2.2. Animal Movement Data

The SENASAG dataset recorded 374,268 unique animal movements from January 1, 2023, to July 25, 2024, including farm-to-farm and farm-to-slaughterhouse movements. After excluding 28,181 records (7.5%) due to missing information, including (a) zero animals moved, (b) identical origin and destination, (c) movements from or to farms not present in the population database or out-of-state premises, our model analyzed 346,087 valid farm-to-farm and farm-to-slaughterhouse movements, corresponding to 7,806,963 animals. The distribution of movements by species, categorized as farm-to-farm and farm-to-slaughterhouse, is shown in Table 1.

### 2.3. Model Description

We extended our multihost, single-pathogen model to simulate FMD outbreaks and control measures and implemented the MHASpread (version 3.0) (https://github.com/machado-lab/MHASPREAD-model) described elsewhere [14, 17]. The supporting information contains a detailed description of the model methodology and parametrization. Briefly, MHASpread consists of a susceptible-exposed-infected-recovered (SEIR) compartment model in which the population, including bovine, swine, and small ruminants, is divided into compartments. The disease transmission is modeled using species-specific transmission probabilities, reflecting the unique transmission dynamics between species (Table S1) and disease progression across compartments for each species within each farm population (Table S2). The model assumes a homogeneous mixing of species within farms, while considering the movement data for between-farm dynamics of animal movements. Our model accounts for movements from farms to slaughterhouses, where transported animals are permanently removed from the simulation. The between-farm spatial transmission process was modeled as a transmission kernel, defining the probability of disease spread based on the distance between infected and noninfected farms. The spatial transmission kernel likelihood of disease transmission decreases as the between-farm distance increases to a maximum of 40 km [18, 19]. The transmission kernel simplifies the complex dynamics of between-farm disease spread by theoretically covering all forms of transmission within this distance limit [20].

### 2.4. FMD Spread and Control Actions

#### 2.4.1. Initial Conditions

**Figure 2:**
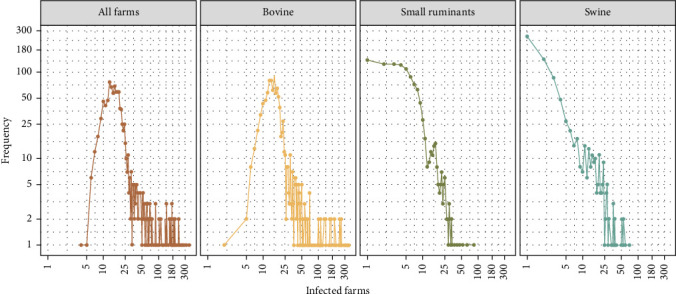
Initial outbreak size. Distribution of the frequencies of an initial number of infected farms by host species in 20 days from initial infection.

A representative sample was drawn from the Bolivian livestock population of 214,109 farms to serve as the initial infected farms. This sample was selected using a multistage, stratified approach, stratifying by species among the nine Bolivian departments. The sample size was calculated with an assumed prevalence of 50% to maximize the total size with a 95% confidence level and a 1.1% margin of error, which results in 1061 sampled farms (geolocation distribution in Figure S1). These selected farms initiated the infection with five infected animals per farm, regardless of the farm's population size. The species targeted for seeding infection was bovine; however, small ruminants were initially infected if no bovines were present on a given farm. Five swine were initially infected if neither bovines nor small ruminants were present. The initial outbreak model was run for 20 days (Figure 2), which generated outbreaks of 1–357 infected farms.

#### 2.4.2. Control Strategies Scenarios

We outline five elimination scenarios based on strategies outlined in the national response plan by SENASAG [21]. These strategies were developed in collaboration with SENASAG animal officials and selected according to local capacities and the organizational structure of the official veterinary service. Briefly, these actions include the depopulation of infected farms, emergency vaccination of infected and uninfected farms, animal movement standstill, trace-back, and the establishment of three control zones around infected farms at the following distances: a 3 km infected zone, a 7 km buffer zone, and 15 km surveillance zone (Figure S2, Table 2). Depopulation: this measure is carried out within infected zones in which infected farms are depopulated, we impose a maximum number of farms that can be eliminated on a daily basis (Table 1), with larger farms being depopulated first. Once a farm was depopulated, its entire animal population was removed, and the farm(s) were excluded from the simulations. Once depopulation's daily capacity is reached, not depopulated farms are scheduled to be depopulated the following day (Table 1). Emergency vaccination was administered in both the infected and buffer zones, which started 15 days after the initial detection and start of control actions to account for the preparation time for the vaccines. Depending on the scenario, infected farms can be vaccinated or not. Emergency vaccinations were administered in both the infected and buffer zones depending on the scenario configuration (Table 1). Once farms in the infected zone are vaccinated, any remaining vaccine doses are used to immunize farms in the buffer zone. If a farm can not be vaccinated within a day due to capacity constraints, it is scheduled for vaccination the following day(s) (Table 1). In each simulation, we randomly chose a vaccine efficacy level. This level determined the percentage of vaccinated animals that became immune to FMD within 15 days, with the efficacy levels being 80%, 90%, or 100% [22, 23]. Refer to the “Vaccination” section in the Supporting Information for more detailed information on the vaccination methodology.

Control measures were initiated on day 20 after the outbreak onset, for the initial day of control actions it was assumed that a 10% proportion of farms would be detected. For example, if 100 farms were infected, 10 were detected, and when fewer than one farm was infected, the model rounded to one detected farm. For the following days of control actions, the detection rate varied based on the number of farms within the control zone(s) and the total number of infected farms. When fewer farms remain under surveillance, the proportion of infected farms within this population increases, making detection more likely (Figure S3). In contrast, when more farms are under surveillance, but the infection is more dispersed, detection may take longer due to lower disease prevalence across the monitored farms (Supporting Information and Figure S3).

We further classified outbreaks into complete and incomplete outbreak elimination, named “controlled” if all infected farms were eliminated within 100 days of the start of control actions. Simulation(s) was deemed “not controlled” if either of the following occurred: (1) the number of infected farms surpassed 400 at any point, or (2) control measures remained active for over 100 days, while more than one farm was still infected.

### 2.5. Spatial Spread Analysis and Mapping

Using the simulation results, we quantified how often each farm was infected across all simulations, regardless of the control scenarios. We then grouped these results by municipality by summing the infections for each farm and calculating the percentage of infected farms relative to the total number of farms in each municipality.

### 2.6. Number of Vaccinated Animals

We calculated the daily number of vaccinated animals; vaccination is used in infected and buffer zones, but not in surveillance zones. We assumed the vaccine produced protection for 6 months with a single dose [23] and vaccinated animals remained alive and nonsusceptible until the end of their production cycle under the vaccinate-to-live strategy scenarios 1, 2, 4, and 5 (Table 2). In our model, depopulation is scheduled before vaccination, meaning that farms that have already been depopulated are no longer considered for immunization. However, infected farms are eligible for vaccination in scenarios 2, 4, and 5. In these cases, animals are counted as vaccinated, even if the farm is depopulated the following day (Table 2).

### 2.7. Software

The language software used to develop the MHASpread model [17] and create graphics, tables, and maps was *R* version 4.2.3 [24] and Python version 3.8.12, *R* utilizing the following packages, sampler [25], tidyverse [26], sf [27], doParallel [28], lubridate [29]; and Python version 3.8.12 with the following packages, Numpy [30], Pandas [31], and SciPy [32].

## 3. Results

### 3.1. Distribution of the Number of Infected Farms Over Time

**Figure 3:**
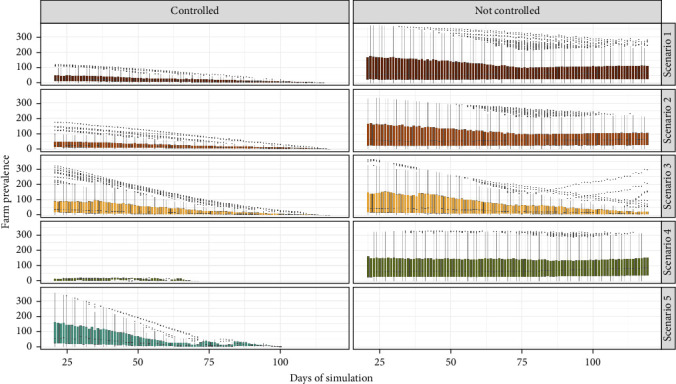
Box plot of epidemic trajectories by control scenarios. Each card represents the distribution of outbreaks that were controlled and those that were not controlled by the different control scenarios. The *y*-axis denotes the number of infected farms, while the *x*-axis represents the simulation days.

Figure 3 represents the outbreak distributions' results over time, distinguishing between “controlled” and “not controlled” outbreaks. Here, scenario 5 eliminated all outbreaks with a median outbreak duration of 3 days, with a maximum of 79 days. In contrast, scenario 4, with a lower vaccination coverage and no depopulation eliminated 0.6% of the outbreaks and the median outbreak duration of 98 days. Both scenario 1 and scenario 2 demonstrated similar effectiveness, controlling 60.3% –62.9% of outbreaks, respectively. However, in scenario 2, the median time to eliminate outbreaks was 9 days less than in scenario 1. Scenario 3, the scenario in which vaccination was not used and 3–5 farms were depopulated daily was effective, controlling 56.76% of outbreaks; nevertheless, it had a significantly longer median outbreak duration of 63 days (Figure 3 and Table 3).

### 3.2. Spatial Spread Analysis and Mapping

**Figure 4:**
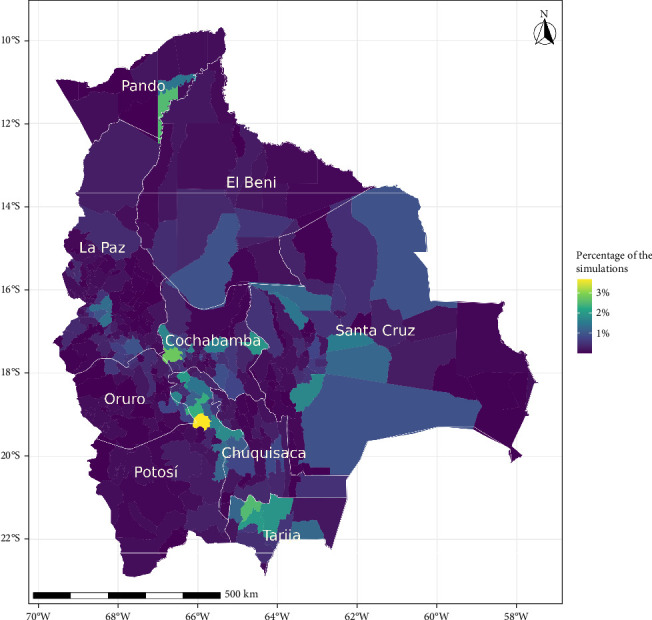
Percentage of infected farms by the municipality. The color represents the percentage of infected farms over all simulations at the municipality level. The gray lines represent the department of Bolivia.

Figure 4 illustrates the percentage of infected farms by municipality. The municipalities with the highest infection rates were Tinguipaya (3.69%), Tapacarí (2.78%), and Méndez (2.57%), while, Detosí, Cochabamba, and La Paz department regions accounted for 25.3%, 23.5%, and 18.6% of these infections, respectively. Santa Cruz and Tarija also experienced notable percentages, with 17.4% and 10.9%, followed by Chuquisaca (5.45%), Pando (4.77%), Oruro (4.33%), and Beni (4.02%).

### 3.3. Number of Vaccinated Animals

**Figure 5:**
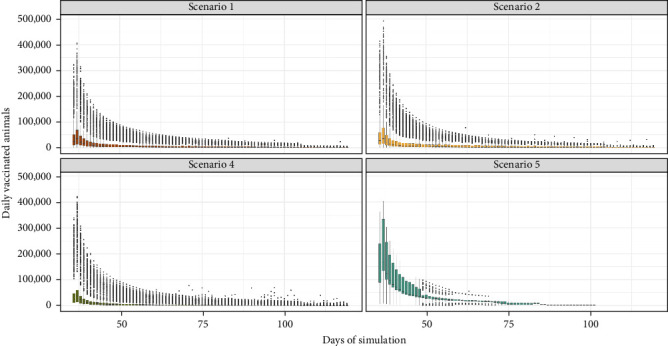
Distribution of the daily number of vaccinated animals by scenario over time.

Figure 5 presents the daily distribution of vaccinated animals over time. The median number of daily vaccinated animals was 1962 (IQR: 726–6752), with daily values ranging from 0 to 492,492. Scenario 5 exhibited the highest daily vaccinated animals with a median of 42,705 (IQR: 22,414–97,500), while scenario 1 showed the lowest median value at 2564 (IQR: 1020– 9608). Both scenarios 2 and 4 yielded intermediate results, with a daily median of 2832 (IQR: 1040–10,380) and 1424 (IQR: 528 –4320), respectively (Table 3 and Figure 5).

## 4. Discussion

In the present study, we used our MHASpread model framework [14, 33] to simulate FMD outbreaks in Bolivia to estimate the outbreak size, map the spatial distribution of the outbreaks and estimate the number of vaccinated animals.

We demonstrated that combining vaccination and depopulation is the most effective strategy for controlling outbreaks, eliminating up to 100% of outbreaks within a median duration of 3 days. In contrast, single-control approaches, depopulation alone controlled only 56.76% of outbreaks with a median duration of 63 days. Low vaccination coverage without depopulation controlled just 0.6% of outbreaks, with the longest median duration of 98 days. Depopulation was the most effective measure in reducing outbreaks, while emergency vaccination reduced the time spent on control actions by vaccinating in a median between 236,526 and 925,338 bovines in 100 days of simulated control actions.

Regions with a high livestock density were correlated with our simulated epidemic hotspots (Figure 4). Understanding the impact of initially infected farms and quantifying the number of affected farms could be used to assess how changes in production patterns may influence future disease dissemination risks. Our results indicate that the regions of Detosí, Cochabamba, and La Paz accounted for 25.3%, 23.5%, and 18.6% of these infections, respectively. This suggests that host density may play an important, particularly significant role in mediating the severity of epidemics in Bolivia [34, 35]. Thus, our results indicated that for regions with higher farm densities, such as the central part of Bolivia, Potosí, Cochabamba, and La Paz (Figure 1), would be ideal locations for stockpiling the necessary resources to respond to a future FMD introduction. These findings align with previous studies showing a correlation between higher farm density and increased infection rates [15, 35–38].

We demonstrated that vaccination and depopulation of infected farms were the most impactful control actions and are part of Bolivia's contingency plans [21]. Vaccinating between 50 and 90 farms and depopulating from 6 to 7 farms daily eliminated 100% of the outbreaks within 3 days of the initial detection. However, this scenario required a median of 255% more vaccinated animals, which is about more than three times as many as any other simulated scenario. In contrast, a scenario with only vaccination eliminates 1% of the outbreaks, highlighting that vaccination alone is insufficient to control most outbreaks under simulated conditions. Moreover, in scenarios where the depopulation rate remained constant for the 100 days of simulated control action (1 to 2 farms/day) and included vaccination, our results indicated that increasing the vaccination rate from 50–90 to 90–120 farms daily reduced the median duration of the outbreak by 9 days demonstrating the benefits of emergency vaccination when combined with depopulation of infected farms, this is because vaccinated animals are less likely to become infectious, reflecting an immune response that reduces virus shedding [39] and provides some level of protection to unvaccinated animals, as the spread of the disease is significantly reduced [40, 41]. Other studies found similar patterns, indicating that vaccination may be effective in reducing the duration and size of FMD outbreaks [42–46]. Indeed, our findings are aligned with studies from Australia, Canada, New Zealand, and the United States, where emergency vaccination was linked to a reduction in the number of infected farms and a shorter outbreak duration [47–51].

We remark that the limitation of simulating FMD dynamics without historical outbreaks limits our ability to perform model calibration and rely on taking parameters from the literature [14]. Furthermore, future work will estimate the cost of all these interventions, which the authors consider essential for designing decision-making tools.

## 5. Conclusion

Our model indicated that vaccination and depopulation would be required to eliminate FMD if reintroduced in Bolivia and the total number of vaccinated animals could reach more than 4,811,808 along with depopulation. Increasing the number of vaccinated farms from 90 to 120, while maintaining depopulation at 2 farms/day improved the percentage of controlled outbreaks by 2%, from 60.3% to 62.9%. In contrast, increasing the depopulation rate to 5 farms/day without vaccination resulted in 56.8% of controlled outbreaks, only 4% less effective than combined strategies involving vaccination and depopulation. These findings highlight that while vaccination contributes to outbreak control, its effectiveness is limited without concurrent depopulation. Ultimately, the likelihood of FMD eradication depends on sufficient vaccine doses, timely deployment, and the capacity to depopulate one to two farms daily, regardless of population size. Early detection and rapid implementation of control measures remain critical for successful containment.

## Figures and Tables

**Table 1:** Number of farm-to-farm and farm-to-slaughterhouse movements and animals count by host species in Bolivia from January 1, 2023, to July 25, 2024.

Species	Movement type	Number of movements	Movements (%)	Number of animals	Animals (%)
Bovine	Farm-to-farm	156,708	95.85	3,738,579	83.66
Small ruminants	Farm-to-farm	3219	1.97	146,726	3.28
Swine	Farm-to-farm	3557	2.18	582,756	13.05
All species	Farm-to-farm	163,484	100.00	4,468,061	100.00
Bovine	Farm-to-slaughterhouse	150,053	82.19	1,759,277	52.72
Small ruminants	Farm-to-slaughterhouse	65	0.04	408	0.01
Swine	Farm-to-slaughterhouse	32,485	17.79	1,579,217	47.27
All species	Farm-to-slaughterhouse	182,603	100.00	3,338,902	100.00

**Table 2:** Description of elimination scenarios and parameters.

Parameter	Scenario 1	Scenario 2	Scenario 3	Scenario 4	Scenario 5
Initial conditions					
Infected farm	Random	Random	Random	Random	Random
The initial simulation day	Random	Random	Random	Random	Random
Detection					
Days of control action	120	120	120	120	120
Control zone(s) radii in kilometers					
Infected zone	3	3	3	3	3
Buffer zone	5	5	5	5	5
Surveillance zone	7	7	7	7	7
Movement restriction					
Standstill (days)	30	30	30	30	30
The standstill in the infected zone	F	F	F	T	F
The standstill in the buffer zone	F	F	F	T	F
The standstill in the surveillance zone	T	T	T	T	T
Direct contact with infected/detected farms	F	F	F	F	F
Traceback duration	1	1	1	2	1
Depopulation					
Limit of farms depopulated per day	1–2	1–2	3–5	0	6–7
Depopulation in the infected zone	F	F	F	F	F
Only depopulate infected farms	T	T	T	T	T
Vaccination					
Days to achieve immunity	15	15	NA	15	15
Number of farms vaccinated in the buffer zone per day	25–45	45–60	NA	25–45	25–45
Number of farms vaccinated in the infected per day	25–45	45–60	NA	25–45	25–45
Proportion of vaccine efficacy	0.8, 0.9, 1	0.8, 0.9, 1	NA	0.8, 0.9, 1	0.8, 0.9, 1
Vaccination of swine	F	F	NA	NA	F
Vaccination of bovines	T	T	NA	T	T
Vaccination of small ruminants	F	F	NA	NA	F
Vaccination in the infected zone	T	T	NA	NA	T
Vaccination in the buffer zone	F	T	NA	NA	F
Vaccination delay (days)	15	15	NA	NA	15
Vaccination in infectious farms	F	T	NA	T	T

*Note:* “T” denotes true, indicating that the control action parameter is applied, and “F” is false when control actions were not applied. “NA” signifies not applicable, meaning the parameter configuration is irrelevant to the current scenario.

**Table 3:** Performance metrics of simulation scenarios for disease control.

Scenario	Vaccination rate/day (infected + buffer zone)	Depopulation rate/day	Percentage of controlled outbreaks (%)	Control actions duration (median [IQR, max])	Total number of vaccinated animals (median [IQR, max])
1	50–90	1–2	60.3	37 (IQR: 11–98, max: 98)	236,526 (IQR: 96,777–468,004, max: 4,676,797)
2	90–120	1–2	62.9	28 (IQR: 11–98, max: 98)	236,389 (IQR: 99,081–486,604, max: 5,016,391)
3	0	3–5	56.8	63 (IQR: 29–97, max: 98)	—
4	50–90	0	0.6	98 (IQR: 98–98, max: 98)	260,305 (IQR: 140,312–445,072, max: 4,811,808)
5	50–90	6–7	100	3 (IQR: 2–6, max: 79)	925,338 (IQR: 404,322–1,889,406, max: 3,691,642)

*Note:* The metrics include the daily vaccination rate, depopulation rate, percentage of controlled outbreaks, the median duration of control actions (including interquartile range and maximum values), and the total number of vaccine animals administered.

## Data Availability

The data that support the findings of this study are not publicly available and are protected by confidential agreements, therefore, are not available.

## Supporting Information

Supporting Information Table S1. The distribution of each host-to-host transmission coefficient (*β*) per animal-1 day-1. Table S2. The within-farm distribution of latent and infectious FMD parameters for each species. Figure S1. The grid density of the sampled farms to seed initial outbreaks. Figure S2. Control zones mapping. Figure S3. Percentage of detected farms according to prevalence and population size.
