# Supplementary material for: Foot-and-Mouth Disease in Bolivia: Simulation-Based Assessment of Control Strategies and Vaccination Requirements
Source: Transbound Emerg Dis. 2025 Sep 25;2025:9055612. doi: 10.1155/tbed/9055612 (PMC12490914; doi:10.1155/tbed/9055612)
Supplement: Supporting Information — Table S1. The distribution of each host-to-host transmission coefficient (β) per animal-1 day-1. Table S2. The within-farm distribution of latent and infectious FMD parameters for each species. Figure S1. The grid density of the sampled farms to seed initial outbreaks. Figure S2. Control zones mapping. Figure S3. Percentage of detected farms according to prevalence and population size. [file 9055612.f1.pdf]

Simulating foot-and-mouth dynamics and control in Bolivia

Model formulation and description

We developed a transmission model that integrates multiple hosts and a single pathogen at different scales to replicate the trajectories of FMD epidemics (Garira, 2018; Cespedes Cardenas and Machado, 2024; Cespedes Cardenas et al., 2024) and allows for simulating countermeasures. This model resulted in the creation of a software package named "MHASpread: A multi-host animal spread stochastic multilevel model" (version 3.0.0), which is available for more detailed information at https://github.com/machado-lab/MHASPREAD-model. MHASpread enables the explicit definition of transmission probabilities specific to each species and the periods when the disease can be transmitted to multiple species. At the level of individual farms, the model accounts for each species' birth and death data.

Within-farm dynamics

For the within-farm dynamics, we assume populations were homogeneously distributed. Species were homogeneously mixed in farms with at least two species, meaning that the probability of contact among species was homogeneous regardless of/when species were segregated in barns and/or paddocks (e.g., commercial swine farms are housed in barns with limited changes of direct contact with cattle). The within-farm dynamics consist of mutually exclusive health states (i.e., an individual can only be in one state per discrete time step) for animals of each species (bovines, swine, and small ruminants). Health states (hereafter, “compartments”) include susceptible (S), exposed (E), infectious, (I), and recovered (R), defined as follows:

Susceptible: animals that are not infected and are susceptible to infection.

Exposed: animals that have been exposed but are not yet infected.

Infectious: infected animals that can successfully transmit the infection.

Recovered: animals that have recovered and are no longer susceptible.

Our model considers birth and death, which is used to update the population of each farm. The total population is calculated as $N = S + E + I + R$. The number of individuals within each compartment transitions from $S\beta \rightarrow\ E, \frac{1}{\sigma} \rightarrow\ I, \frac{1}{\gamma} \rightarrow\ R$ according to the following equations:

$$\frac{dS_i(t)}{dt} = u_i(t) - v_i(t) - \frac{\beta S_i(t) I_i(t)}{N_i} \quad (1)$$

$$\frac{dE_i(t)}{dt} = \frac{\beta S_i(t) I_i(t)}{N_i} - v_i(t) E_i(t) - \frac{1}{\sigma} E_i(t) \quad (2)$$

$$\frac{dI_i(t)}{dt} = \frac{1}{\sigma} E_i(t) - \frac{1}{\gamma} I_i(t) - v_i(t) I_i(t) \quad (3)$$

$$\frac{dR_i(t)}{dt} = \frac{1}{\gamma} I_i(t) - v_i(t) \quad (4)$$

Transmission depends on infected and susceptible host species, as reflected by the species-specific FMD transmission coefficient β (Table S1).

Supplementary Table S1. The distribution of each host-to-host transmission coefficient (β) per animal^{-1} day^{-1}.

**Infected species**	**Susceptible taxon**	**Transmission coefficient (β), shape and distribution (min, mode, max)**	**Reference**
Bovine	Bovine	PERT (0.18, 0.24, 0.56)	Calculated from the 2000-2001 FMD outbreaks in the state of Rio

			Grande do Sul (da Costa et al., 2022)
Bovine	Swine	PERT (0.18, 0.24, 0.56)	Assumed
Bovine	Small ruminants	PERT (0.18, 0.24, 0.56)	Assumed
Swine	Bovine	PERT (3.7, 6.14, 10.06)	Assumed (Eblé et al., 2006)
Swine	Swine	PERT (3.7, 6.14, 10.06)	(Eblé et al., 2006)
Swine	Small ruminants	PERT (3.7, 6.14, 10.06)	Assumed (Eblé et al., 2006)
Small ruminants	Bovine	PERT (0.044, 0.105, 0.253)	Assumed (Orsel et al., 2007)
Small ruminants	Swine	PERT (0.006, 0.024, 0.09)	(Goris et al., 2009)
Small ruminants	Small ruminants	PERT (0.044, 0.105, 0.253)	(Orsel et al., 2007)

Bolivian data do not consistently record the number of animal births and deaths, (excluding those sent to slaughterhouses). Despite this limitation, our model can incorporate these births and deaths whenever data are available. Here, births are represented by the number of animals born alive $u_i(t)$ that enter the S compartment on the farm i at the time t according to the day-to-day records; similarly, $v_i(t)$ represent the exit of the animals from any compartment due to death at

the time t. The transition from E to I is driven by $1/\sigma$, and the transition from I to R is driven by $1/\gamma$; these values are drawn from the distribution generated from each specific species according to the literature (Supplementary Material Table S2).

Supplementary Table S2. The within-farm distribution of latent and infectious FMD parameters for each species.

FMD parameter	Species	Mean, median (25th, 75th percentile) in days	Reference
Latent period, σ	Bovine	3.6, 3 (2, 5)	(Mardones et al., 2010)
	Swine	3.1, 2 (2, 4)	(Mardones et al., 2010)
	Small ruminants	4.8, 5 (3, 6)	(Mardones et al., 2010)
Infectious period, γ	Bovine	4.4, 4 (3, 6)	(Mardones et al., 2010)
	Swine	5.7, 5 (5, 6)	(Mardones et al., 2010)

Small ruminants	3.3, 3 (2, 4)	(Mardones et al., 2010)

Note: The time unit is days.

Kernel transmission dynamics

Spatial transmission encompasses a range of mechanisms, such as airborne transmission, animal contact over fence lines, and equipment sharing between farms (Boender et al., 2010; Boender and Hagenaars, 2023). Local spread was modeled using a spatial transmission kernel, in which the likelihood of transmission decreased as a function of the between-farm distance. The probability PE at time t describes the likelihood that a farm becomes exposed and is calculated as follows:

$$PE_j(t) = 1 - \prod_i \left(1 - \frac{I_i(t)}{N_i} \varphi e^{-\alpha d_{ij}}\right) \quad (5)$$

where j represents the uninfected population and ad_{ij} represents the distance between farm j and infected farm i, with a maximum of 40 km (Cespedes Cardenas et al., 2024). Given the extensive literature on distance-based FMD dissemination and a previous comprehensive mathematical simulation study (Björnham et al., 2020), distances above 40 km were not considered. Here, $1 - \frac{I_i(t)}{N_i} \varphi e^{-\alpha d_{ij}}$ represents the probability of transmission between farms i and j scaled by infection prevalence of farm i, $\frac{I_i}{N_i}$, given the distance between the farms in kilometers. The parameters φ and α control the shape of the transmission kernel; $\varphi = 0.044$, which is the probability of transmission when $d_{ij} = 0$, and $\alpha = 0.6$ control the steepness with which the probability

declines with distance (Boender et al., 2010; Boender and Hagenaars, 2023; Cespedes Cardenas et al., 2024).

FMD spread and control actions

We initially simulated a silent spread over 20 days, generating a broad spectrum of outbreak scenarios before implementing any control measures, as shown in Figure 2. The FMD control scenarios include the following measures: i) depopulation of infected farms, ii) emergency vaccination of farms within the infected and buffer zones, iii) a 30-day standstill on animal movement, and iv) the establishment of three distinct control zones around infected farms: a 3 km infected zone, a 7 km buffer zone, and a 15 km surveillance zone (Supplementary Material Figure S3).

The depopulation of infected farms involves destroying all animals from farms within the infected zone(s), with priority given to farms that have larger animal populations. Once depopulated, these farms are excluded from the simulation. When the daily depopulation capacity cannot accommodate all infected farms in one day, the remaining farms are scheduled for depopulation the following day or as soon as possible, subject to each simulated scenario's capacity limits (Table 2).

Vaccination: Bovine farms within the infected and buffer zones received emergency vaccination 15 days after establishing the control zone. Due to capacity constraints, farms unable to be vaccinated within a day were vaccinated on subsequent days (s).

The number of farms vaccinated per day depends on the scenario being simulated (Table 2). Vaccination is administered to farms within the infected and/or buffer zones, following predefined criteria, with priority given to farms with the largest population. Each farm's

vaccination begins on a specified day, with animals progressively transferred from the S, E, I, and R compartment to the V compartment at a daily rate proportional to the remaining population in each compartment. The transfer is based on the vaccination rate and vaccine efficacy, with a maximum vaccination protection of 90% that was achieved within 15 days

The total number of animals eligible for vaccination is given by:

$$n = (1 - ve) * N \tag{6}$$

where ve represents the vaccine efficacy and N is the total number of cattle on the vaccinated farm. From the eligible to be vaccinated population, the number of animals moved to the vaccinated status each day is drawn from a binomial distribution:

$$X \sim Binomial(n, p)$$

where the probability p is defined as:

$$p = \frac{1}{vt}$$

Here, vt represents the time required for the full progression of immunity following vaccination (15 days), assuming that by the end of this period, all eligible animals will have developed immunity.

Traceability: We employed contact tracing to identify farms that had direct contact with infected farms in the past 30 days. These farms were subject to surveillance, including clinical examinations and detection procedures. Farms that tested positive during contact tracing were classified as detected infected farms.

Infected farms detection: We assumed that 10% of the infected farms were detected at the start of control measures, which occurred 10 days after the introduction of the index case. For example, if 100 farms were infected initially, 10 farms were detected. If the number of detected farms was less than one, we rounded up to one detected farm.

The detection rate was influenced by two main factors: the total number of farms within the control zones and the number of infected farms. For instance, when fewer farms were under surveillance but there was a higher number of infected farms, the likelihood of detection increased (Supplementary Material Figure S3). Infected farms outside the control zones were also included among those subjected to detection.

The probability of detecting a new infected farm P_i considers the number of infected farms and the total number of farms under surveillance.

-P_i : Number of farms in surveillance.

- I_i: Number of infected farms.

- E: Number of farms found in the current iteration

The algorithm operates as follows:

1. If $I_i < 5$, E is set to a random value between 0 and I_i (inclusive), i.e.,$E = sample(0{:}\ I_i, 1)$.

2. If $Ii \geq 5$ a probability distribution is calculated using the hypergeometric distribution with parameters ($m = I_i$), ($n = P_i$) and ($k = \frac{p_i}{3}$).

Sample a value p from this probability distribution.

Calculate $E = [I_i * p]$

If $E = 0$, set $E = 1$

Traceability and movement standstill: We employed contact tracing to detect farms with direct links to infected farms within the past 30 days, subjecting these farms to surveillance. Positive farms from trace back were categorized as detected infected farms, triggering the application of the same criteria for control zones. In addition, a 30-day restriction on animal movement was enforced across all three control zones, prohibiting both incoming and outgoing movements. The control zones remained in place, and the movement standstill was maintained until depopulation efforts were fully completed.

References

Björnham, O., R. Sigg, and J. Burman, 2020: Multilevel model for airborne transmission of foot-and-mouth disease applied to Swedish livestock. (Bryan C. Daniels, Ed.)*PLOS ONE* **15**, e0232489, DOI: 10.1371/journal.pone.0232489.

Boender, G.J., and T.J. Hagenaars, 2023: Common features in spatial livestock disease transmission parameters. *Sci. Rep.* **13**, 3550, DOI: 10.1038/s41598-023-30230-w.

Boender, G.J., H.J.W. van Roermund, M.C.M. de Jong, and T.J. Hagenaars, 2010: Transmission risks and control of foot-and-mouth disease in The Netherlands: Spatial patterns. *Epidemics* **2**, 36–47, DOI: 10.1016/j.epidem.2010.03.001.

Cespedes Cardenas, N., F. Amadori Machado, C. Trois, V. Maran, A. Machado, and G. Machado, 2024: Modeling foot-and-mouth disease dissemination in Rio Grande do Sul, Brazil and evaluating the effectiveness of control measures. *Front. Vet. Sci.* **11**.

Cespedes Cardenas, N., and G. Machado, 2024: MHASPREAD model [Online] Available at https://github.com/machado-lab/MHASPREAD-model (accessed September 16, 2024).

da Costa, J.M.N., L.G. Cobellini, N.C. Cardenas, F.H.S. Groff, and G. Machado, 2022:

Assessing epidemiological parameters and dissemination characteristics of the 2000 and 2001 foot-and-mouth disease outbreaks in Rio Grande do Sul, Brazil. *bioRxiv*2022.05.22.492961, DOI: 10.1101/2022.05.22.492961.

Eblé, P., A. De Koeijer, A. Bouma, A. Stegeman, and A. Dekker, 2006: Quantification of within- and between-pen transmission of Foot-and-Mouth disease virus in pigs. *Vet. Res.* **37**, 647–654, DOI: 10.1051/vetres:2006026.

Garira, W., 2018: A primer on multiscale modelling of infectious disease systems. *Infect. Dis. Model.* **3**, 176–191, DOI: 10.1016/j.idm.2018.09.005.

Goris, N.E., P.L. Eblé, M.C.M. de Jong, and K.D. Clercq, 2009: Quantifying foot-and-mouth disease virus transmission rates using published data. *ALTEX - Altern. Anim. Exp.* **26**, 52–54, DOI: 10.14573/altex.2009.1.52.

Mardones, F., A. Perez, J. Sanchez, M. Alkhamis, and T. Carpenter, 2010: Parameterization of the duration of infection stages of serotype O foot-and-mouth disease virus: an analytical review and meta-analysis with application to simulation models. *Vet. Res.* **41**, 45, DOI: 10.1051/vetres/2010017.

Orsel, K., A. Dekker, A. Bouma, J.A. Stegeman, and M.C.M. De Jong, 2007: Quantification of foot and mouth disease virus excretion and transmission within groups of lambs with and without vaccination. *Vaccine* **25**, 2673–2679, DOI: 10.1016/j.vaccine.2006.11.048.

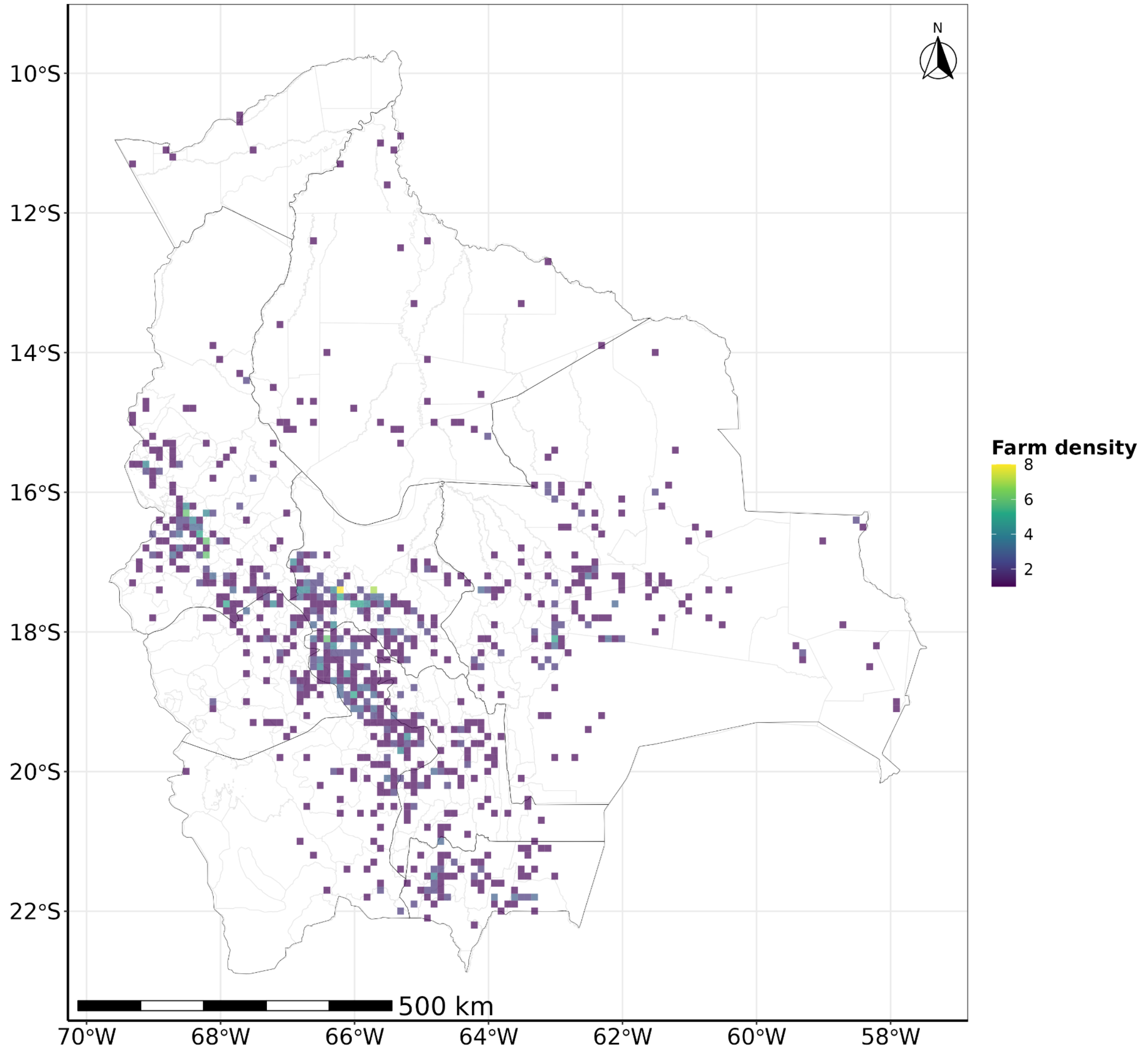

Supplementary Figure S1. The grid density of the sampled farms to seed initial outbreaks. A 10 km^2 grid was projected onto the map, and the density of pig premises was represented as the number of premises allocated in each grid cell. Black lines represent the political state division of Bolivia. The gray lines represent the political municipal division of Bolivia.

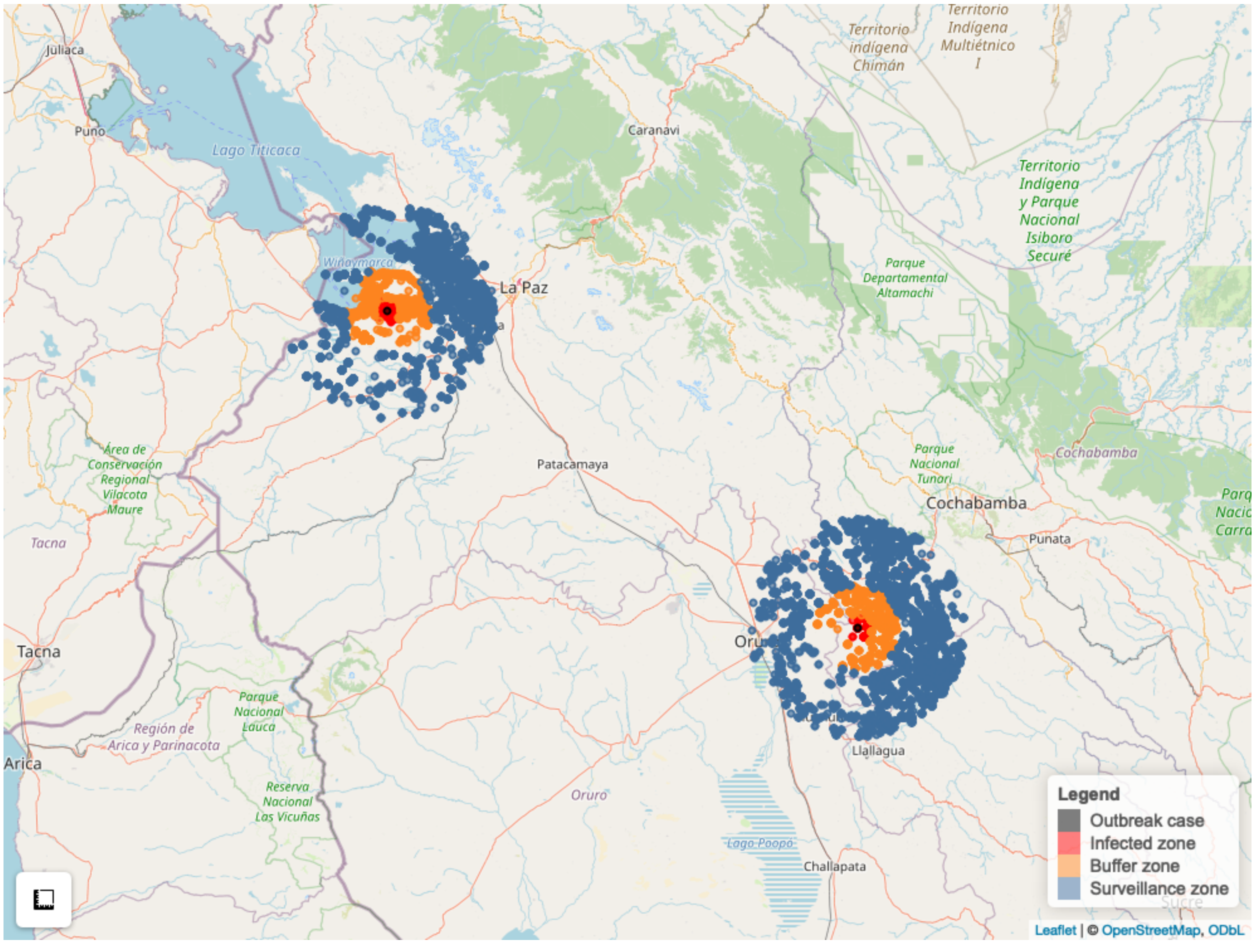

Supplementary Figure S2. Control zones mapping. The infected control zones of 3 km are represented as red dots, the 7 km buffer zone is represented as orange dots, and the 15 km surveillance zone is represented as blue dots.

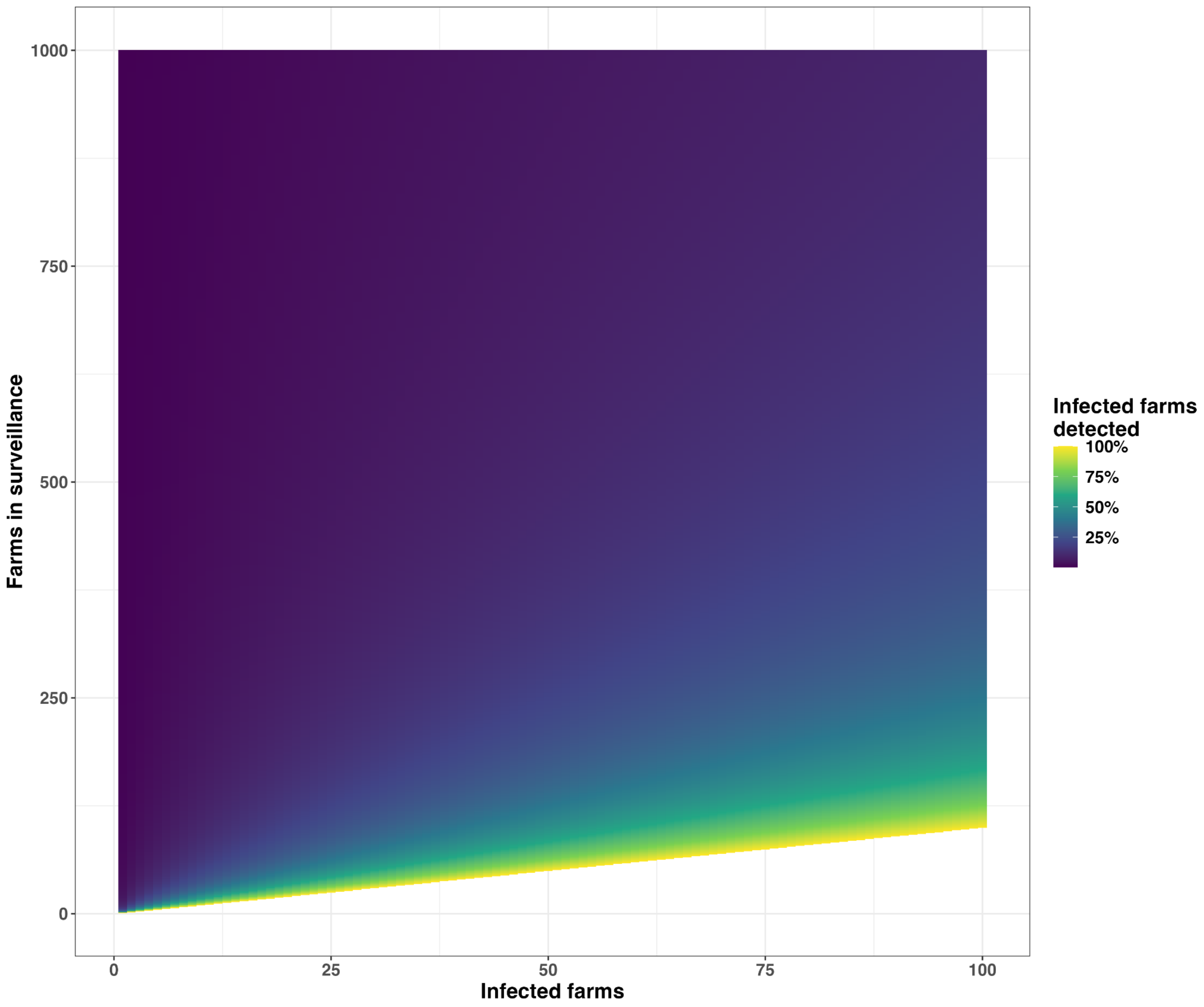

Supplementary Figure S3. Percentage of detected farms according to prevalence and population size. The y-axis represents the number of farms under surveillance, while the x-axis represents the number of infected farms in the population. The color represents the percentage of infected farms that will be detected. Figure from (Cespedes Cardenas et al., 2024).
